# Designing of an Enhanced Fuzzy Logic Controller of an Interior Permanent Magnet Synchronous Generator under Variable Wind Speed

**DOI:** 10.3390/s23073628

**Published:** 2023-03-30

**Authors:** Uossif Mohamed Matoug Masoud, Pratibha Tiwari, Nishu Gupta

**Affiliations:** 1 Department of Electrical Engineering, Sam Higginbottom University of Agriculture, Technology and Sciences, Prayagraj 211007, India; 2 Department of Electronic Systems, Norwegian University of Science and Technology, 2821 Gjøvik, Norway

**Keywords:** enhanced fuzzy logic controller (EFLC), interior permanent magnet synchronous generator (IPMSG), sensors, torque ripple, Weibull distribution-based chicken swarm optimization (WDCSO), wind energy conservation system (WECS)

## Abstract

On account of active governmental stimulation operations in many countries, the residential production of electricity from renewable resources has increased considerably. Due to high efficiency and reliability, a recommended solution for residential wind energy conservation systems (WECS) is permanent magnet synchronous generators (PMSG). A higher torque ripple (TR), engendered by the contact of the stator with the rotor’s magnetomotive force harmonics, is one foremost issue in PMSGs. To control the synchronous generator, numerous control schemes have been proposed. However, it still faces a challenge in the diminishment of the TR. An enhanced fuzzy logic controller (EFLC) in interior PMSG (IPSMG) under variable wind speed (WS) has been proposed in this article to address this challenge. Initially, the wind turbine (WT) system was designed, and the IPMSG was proposed. A hysteresis controller (HC) and fuzzy logic controller (FLC) are the two controller types utilized in this model to control TR. This methodology used the EFLC to eliminate errors during the control. By using the proper membership function (MF) for boundary selection in the WDCSO algorithm, an enhancement was executed. Better performance in TR reduction was attained by the proposed model grounded in the analysis.

## 1. Introduction

Nowadays, owing to their huge torque density along with high efficacy, PMSGs are prominent in the industry’s electrical drive model [1]. When analogized with induction generators, superior characteristics, such as diminished copper loss yielding higher power density and higher efficacy along with a reduction in size and weight, are shown by PMSGs [2]. IPM electric machines are utilized for numerous applications, such as industrialized applications, domestic appliances, and electric and hybrid vehicles. Because of their high efficacy, power, and torque density, they are well known [3,4].

For megawatt-level WT producers, inconsistent speed wind power models utilizing PMSGs and gearless drive trains combined with full-scale power converters have attained major consideration in the previous few years [5]. As expected, a large TR affects the inset PMSG that confines the PMSG in high-precision applications [6]. Mechanical resonance’s excitation on the load side and speed oscillation, particularly for large-performance applications, along with acoustic noise are the major outcomes of TRs [7]. Cogging torque (CT), PM torque, and reluctance torque are measured as the sources affecting the TR [8].

Relying on the WS to attain the necessary shaft speed, various control strategies have been formulated. For medium WECS, a high cost combined with diminished reliability is unavoidable [9]. Existing globally, wind energy has been highly approved by governments, leading to wind farm development’s growth [10]. More effective models of diminished ripple reduction for PMSGs have been largely investigated in the previous decades [11]. Rotor step-skewing with sinusoidal profiling of the rotor surface is the typical and prominent technique. The diminishment of the maximal torque in the sensorless drive and enhancements in the generator cost are the limitations of these models [12]. The WECS measurements of diverse system quantities (e.g., speed, voltage, current, etc.) are requisite for the successful implementation of these control models, which are executed utilizing sensors [13].

To diminish CT, fractional-slot concentrated windings (FSCW) are used in various applications and are often improved under load conditions [14]. Grounded on average torque and ripple, an impact in the rotor geometry on the device’s performance is made by employing a rotor configuration specified by various pole flux barriers [15]. The IPM generator with reluctance torque is the prominent design, along with the total output torque contributed by PM [16]. The TR is abolished to a better level by short-pitching the winding by a few slots to diminish low-order MMF space harmonics, as illustrated in previous research [17]. By the linkage of the stator along with the rotor’s higher-order redundant harmonics, TR is caused [18]. The models used to diminish the TR are the machine-design-based approach and the machine-control-based approach. To lessen the PM flux linkage’s spatial harmonics along with the CT, the machine-design-centered approach is used, focusing on augmenting the stator in combination with rotor modeling. The machine-control-based approach regulates the stator currents to smooth PMSG’s output torque [19]. Still, improvements are requisite for torque reduction. For TR reduction, a novel FLC is proposed here.

The main objective of this work is to simulate and model an IPM synchronous generator. In addition, to solve the problems of the existing research and improve the performance of the torque ripple reduction.

The structure of the presented research is as follows: In Section 2, the existing research associated with TR is given; in Section 3, the proposed research methodology is described; in Section 4, the methodology’s result values are explained; and the work is concluded in Section 5.

## 2. Related Work

The authors of [20] presented a rotor shape optimization grounded on the modeling of notches along with a rotor flux barrier (FB). The consequences of rotor notching on TR along with CT were first discussed. To choose the optimal FB combined with rotor W-notch dimensions in the external rotor blueprint, differential evaluation (DE) optimization was executed. For a W-notch, the optimal values were selected. The benefits of opting for a W-notch in the exterior rotor design were demonstrated when analogized with a benchmark C-notch. To analogize TR with CT outcomes, finite element (FE) simulations were executed in detail by measuring the rotor’s notches. It was observed that the preference for the W-notch appreciably enhances the torque profile in rare-earth. To validate the simulation outcomes, the experiments were executed in prototypes.

The researchers in [21] suggested a TR reduction model of DTC by the FC incorporating an optimal selection strategy of voltage vectors in a five-phase induction generator. Without utilizing the parameter, the DTC was controlled easily. Therefore, by utilizing the designed FC, the voltage vector insertion time was changed by the algorithm. In accordance with the torque error, the optimized voltage vector selection scheme was utilized. The control algorithm’s effectiveness was shown in the simulation outcomes. More than a 30% diminishment in TR was attainable using this model when analogized with the prevailing models.

The researchers in [22] introduced a new arithmetic formula and a framed model to diminish the TR while augmenting the efficacy combined with the interior PMSG’s control performance. Centered on ICF, the advanced inverse cosine function (AICF) was utilized to recognize the asymmetric rotor structure for denoting the sinusoidal air gap flux density distribution, taking into consideration a definite load state. Furthermore, by employing the AICF, the TR and peak value diminished the induced voltage’s total harmonic distortion (THD), and a diminished iron loss was attained when analogized with the prevailing methods. The importance of the low peak value along with the induced voltage’s THD was referred to, as they influence the generator’s control performance. Specifically, in the high-speed region, a higher efficiency was induced by diminished iron loss. The features of eight-pole, twelve-slot generators having dissimilar rotor shapes were examined via finite element analysis (FEA) to validate the designed scheme’s validity.

The researchers in [23] developed a Lyapunov-centered finite control set model for predictive direct torque control (FCS-MPC) meant for the PMSM. For the PMSM’s torque prediction, the two-level converter’s eight voltage vectors employed a finite control. A cost function bearing the torque error and the maximum torque per ampere (MTPA) function combined with the present drawback was presented. To evaluate each voltage vector’s duty cycle analogized to the prevailing FCS-MPC, the cost function’s paramount part was wielded as a Lyapunov function. As for the eight vectors and duty cycles, an optimum voltage was attained via the optimum voltage vector. The control scheme’s performance was validated by the experimental outcomes.

The authors of [24] presented a constant switching frequency (CSF)-centered three-level direct torque control (3L-DTC) algorithm. Below all working situations and operating at a low CSF level, an effective diminish in TR was attained using the algorithm. For the 3L-DTC algorithm, a detailed analysis in addition to design guidelines was presented. Neutral point voltage variations combined with smooth voltage vector switching were the typical issues with the ‘3′ level inverter and were addressed. To validate the model’s effectiveness, the experimental outcomes were presented.

The authors of [25] presented a new scheme to generate stator currents, offering a surface PMSG presenting a stator winding’s asymmetry. To evaluate the current that diminished TR through the asymmetry, a systematic model of SPMSG was formulated. For the torque generation, this model was centered on the amalgamation of the stator and rotor’s flux density space harmonics. To neutralize the harmonic torque constituent at double the supply frequency, this study outlined a defined stator inverse current system. Various supply scenarios were applied in the experimental tests, and it was confirmed that those attained through the analytical study effectively concerned the inverse current’s amplitude.

The existing research methodology gives more ideas for reducing torque ripples of IPMSG, but improvement is still needed because the error presented during the control strategy affected the performance of the system. This research methodology proposes a novel EFLC. The focus of the methodology is to avoid errors and reduce torque ripple.

## 3. Proposed EFLC-Based Torque Ripple Reduction in IPMSG

**Figure 1:**
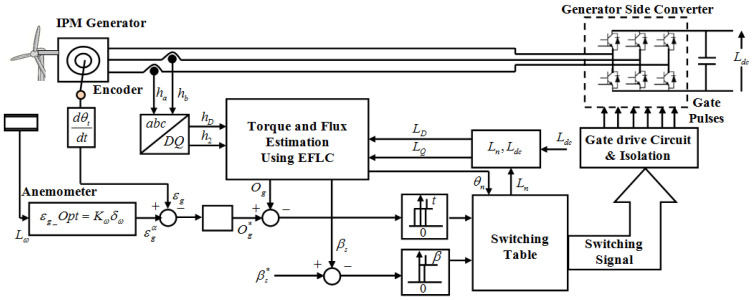
Block diagram for the proposed framework centered on TR reduction.

The wide use of PMSG is attributed to its characteristics of high torque at low speeds. The factor that limits the utilization of PMSG is the TR. The proposed model uses the EFLC to diminish the TR. The WT model is explained initially; then, the IPMSG model is explained; and, following that, the controller’s design is explained. Figure 1 displays the TR reduction in the proposed framework.

### 3.1. Modeling of Wind Turbine

Torque is generated by the WT from wind energy. The torque is transferred from the generator’s shaft to the rotor. An electrical torque is generated by the generator. Determining whether the mechanical system increases, diminishes, or remains at a stable speed is determined by the distinction between the WT’s mechanical torque and the generator’s electrical torque. For charging a DC-link capacitor, the linkage betwixt the generator and the three-phase inverter rectifies the generator’s current coming out. Through a transformer, the DC link offers a second-phase inverter coupled with the grid. To analogize with the grid-side data, the WS data, pitch angle, rotor RPM, and inverter output are established through a control system. By utilizing digital signal processing, the information is resolved to make the perfect signal to manage these mechanisms. The main goals of wind turbines are the synchronization with the utility grid and the exportation of power to it. Regarding air density, the rotor swept area, along with WS, and the quantity of energy transferred by the wind to the rotor were identified. Only accessible wind power parts, along with the actual power extracted by a WT, are captured by the WT rotor blades, which are specified by:

(1) $$W_{T}=EF_{p}\times W_{wind}$$

(2) $$W_{T}=\frac{1}{2}\varpi B\zeta_{w}^{3}\times EF_{p}\left(\psi ,\mu \right)$$

where the turbine power is signified as $W_{T}$, the coefficient of performance is indicated as $EF_{p}$, the Betz limit is a function of tip speed ratio (TSR) (ψ) and pitch angle (μ), the density of air is represented as ϖ, the WS in m/s is indicated as $\zeta_{w}$, and the area in m^2^ is denoted as B. By dividing the rotor tip speed by the wind tip speed, the turbine’s TSR is calculated, which is expressed as:

(3) $$TSR=\varepsilon_{m}H/\zeta_{w}$$

where the rotor’s speed in rad/s is notated as $\varepsilon_{m}$, and the turbine’s radius is denoted by H. When the turbine’s operating speed is high, the maximum power is generated by the turbine. So, maintaining the rotor’s speed at an optimal value is important. By variations in the WS, the rotor speed can be manipulated. The optimum power generated by the WT is given as:

(4) $$K_{opt}=\frac{1}{2}\varpi B\zeta_{p\_opt}{\left(\varepsilon_{m\_opt}H/\psi_{r\_opt}\right)}^{3}$$

(5) $$K_{opt}=\frac{1}{2}\varpi B\zeta_{p\_opt}{\left(H/\psi_{r\_opt}\right)}^{3}$$

where

(6) $$\varepsilon_{m\_opt}=\varepsilon_{g\_opt}=\left(\psi_{r\_opt}/H\right)\;\zeta_{m}=K_{w}\zeta_{w}$$

where the optimum value is specified as $\psi_{r\_opt}$. The optimum torque is calculated as

(7) $$U_{m\_opt}=K_{opt}{\left(\varepsilon_{m\_opt}\left(t\right)\right)}^{2}$$

### 3.2. Modeling of IPMSG

Using the following assumptions, the model of the PMSG devoid of damper winding is designed on a rotor reference frame:

(a) Neglect of saturation.
(b) The induced EMF is sinusoidal.
(c) Negligible losses in Eddy current and hysteresis.
(d) No field current dynamics.

Within the stator reference frame, the synchronous generator model was designed. The machine rotates synchronously with the rotor. To analyze the IPMSG model, the *d*-axis and *q*-axis are utilized as reference signals. The *d*- and *q*-axes voltages are tabulated as:

(8) $$L_{d}=h_{d}\;\;Z_{d}-\upsilon_{r}\beta_{q}+\rho \beta_{d}$$

(9) $$L_{q}=h_{q}\;\;Z_{q}+\upsilon_{r}\beta_{d}+\rho \beta_{q}$$

The flux linkages of *d*- and *q*-axes are tabulated as

(10) $$\beta_{d}=G_{d}\;h_{d}+\beta_{m}$$

(11) $$\beta_{q}=G_{q}\;h_{q}$$

In the voltage equation, the flux linkages are substituted as

(12) $$L_{d}=h_{d}\;\;Z_{d}-\upsilon_{r}G_{q}\;h_{q}+\rho \left(G_{d}\;h_{d}+\beta_{m}\right)$$

(13) $$L_{q}=h_{q}\;\;Z_{q}+\upsilon_{r}\left(G_{d}\;h_{d}+\beta_{m}\right)+\rho G_{q}\;h_{q}$$

where d/dt operator is denoted by ρ, the q,d axes voltages are represented as $L_{q}$ and $L_{d}$, the q,d axes stator currents are signified as $h_{q}$ and $h_{d}$, the q,d axes inductances are denoted as $G_{q}$ and $G_{d}$, the q,d axes stator flux linkages are symbolized as $\beta_{q}$ and $\beta_{d}$, while the stator resistance and rotor speed are indicated by r and $\upsilon_{r}$, respectively, owing to rotor magnets joining the stator $\beta_{m}$ flux linkage. The developed generator torque is given by

(14) $$O_{g}=\frac{3}{2}\left(\frac{P}{2}\right)\left(\beta_{d}h_{q}-\beta_{q}h_{d}\right)$$

(15) $$O_{g}=\frac{3}{2}\left(\frac{P}{2}\right)\left(\beta_{m}h_{q}+\left(G_{d}-G_{q}\right)h_{d}\;\;h_{q}\right)$$

The mechanical torque equation is

(16) $$Q_{e}=Q_{l}+A\upsilon_{m}+R\frac{d\upsilon_{m}}{dt}$$

The rotor mechanical speed is given by

(17) $$\upsilon_{m}=\int \left(\frac{Q_{e}-Q_{l}-A\upsilon_{m}}{R}\right)dt$$

where the friction coefficient is indicated as A, the load torque is modeled as $Q_{l}$, and the moment of inertia is depicted as R. $h_{q}$ and $h_{d}$, in terms of current $I_{m}$, are

(18) $$\left[\begin{array}{l} h_{q}\\ h_{d} \end{array}\right]=\left(I_{m}\right)\left[\begin{array}{l} \sin\;\delta \\ \cos\;\delta \end{array}\right]$$

The electromagnetic torque equation is given by

(19) $$Q_{e}=\frac{3}{2}\left(\frac{P}{2}\right)\left[\frac{1}{2}\left(G_{d}-G_{q}\right)I_{m}^{2}\;\sin2\delta +\beta_{f}I_{m}\;\sin\delta \right]$$

By the following equation, the flux linkage in the stator is described as

(20) $$\left|\beta_{q}\right|=\sqrt{\beta_{D2}-\beta_{Q2}}$$

(21) $$∠\theta =\tan-1\left(\beta_{Q}-\beta_{D}\right)$$

In the stator reference, the electromagnetic torque is tabulated as

(22) $$O_{g}=-3/2\;\left(\beta_{D}\;h_{Q}-\beta_{Q}\;h_{D}\right)$$

Using generator parameters, the mathematical expression for torque is given by

(23) $$O_{g}=\frac{-3P\left|\beta_{s}\right|}{4\;G_{d}\;G_{q}}\left(2\beta_{m}\;G_{q}\;\sin\delta -\left|\beta_{q}\right|\left(G_{d}-G_{q}\right)\;\sin\left(2\delta \right)\right)$$

**Figure 2:**
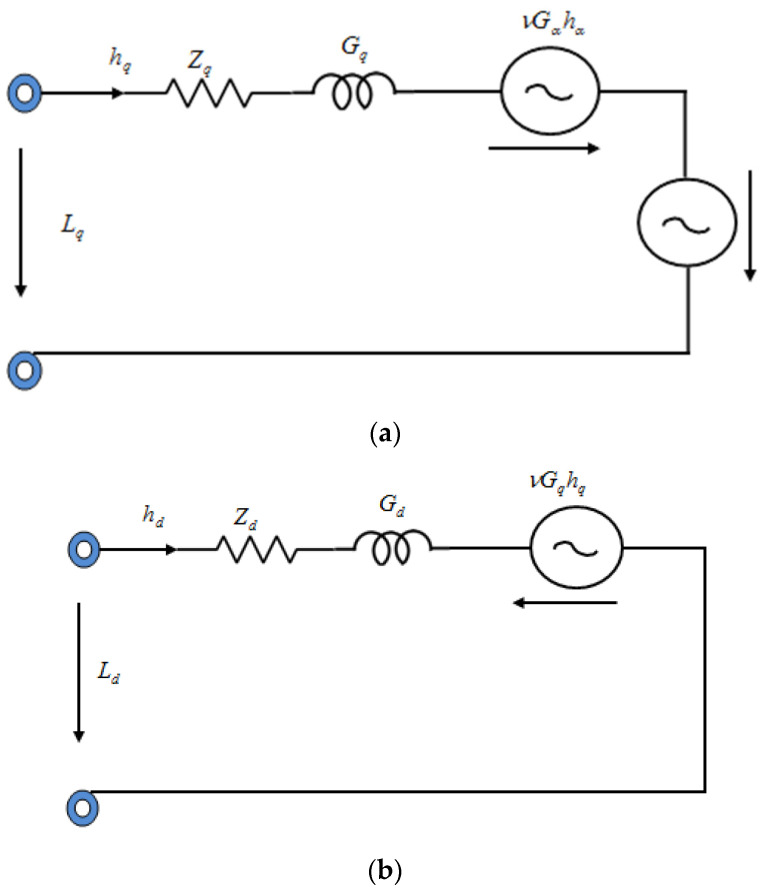
Equivalent circuit for (**a**) *q*-axis, and (**b**) *d*-axis.

In three-phase generators, the stator voltage vector $L_{s}$ in distributed stator windings is given by

(24) $$L_{s}=2/3\;\left(L_{a}-L_{b}\;e_{3}^{j2\pi }+L_{c}\;e_{3}^{j2\pi }\right)$$

where phase ‘a’ is considered as a reference. Under variable wind conditions, a rectifier is connected across the generator to supply constant output across the load. Regarding the *d*- and *q*-axes, the synchronous generator’s equivalent circuit is portrayed in Figure 2.

**Figure 3:**
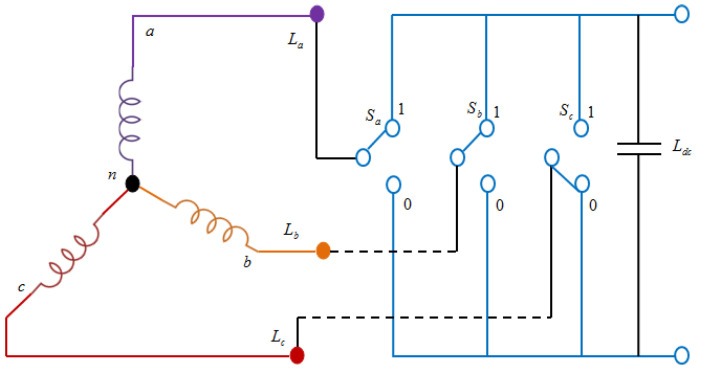
Rectifier connection diagram.

Across the IPM synchronous generator, the bidirectional power electronic switches are connected in the proposed method, as displayed in Figure 3.

**Figure 4:**
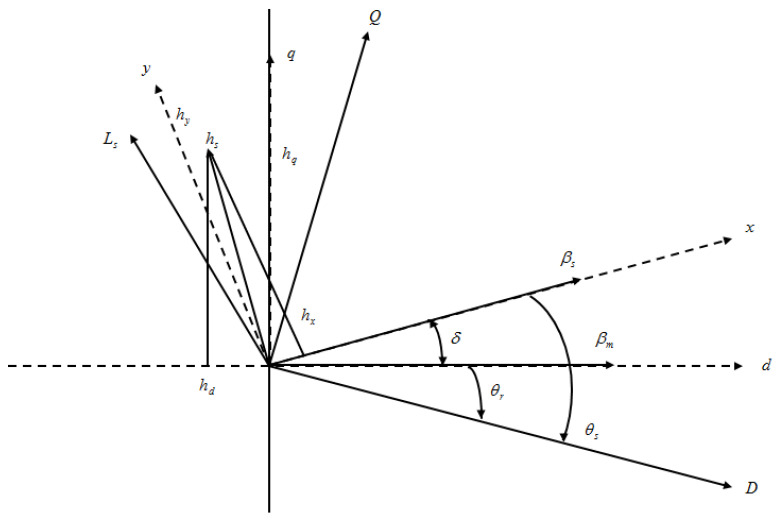
Flux linkages in stator and rotor.

Six voltage vectors together with two zero voltage vectors as shown in Figure 4 are engendered by the rectifier. The six voltage vectors are distanced at 60 degrees apart from each other. The voltage vectors are given as

(25) $$L_{s}\left(S_{a}\;S_{b}\;S_{c}\right)=L_{D}\left(S_{a}-S_{b}\;e_{j2\pi }^{j2\pi }+S_{c}\;e_{3}^{j2\pi }\right)$$

where *L_D_* = $2/3\;\;L_{dc}$ and $L_{dc}$ = DC link voltage.

### 3.3. Controllers

Two hysteresis controllers and an FLC directly regulate the torque and stator flux in this approach. The torque demand is estimated via the FLC.

#### 3.3.1. Hysteresis Controller

For creating the switching pulses, the hysteresis current control method is used. Because of its noncomplex implementation, lack of any tracking error, outstanding stability, quick transient response, inherent restricted maximal current, and intrinsic robustness to load parameter variations, the HCC is the most comprehensively utilized scheme among various current control frameworks. By optimizing the switching nodes, the current and generator’s flux are controlled by the HCs used in the IPMSG. By choosing the voltage space vectors, the desired response of torque along with flux linkage is acquired. Due to the vector position linked to stator flux along with the HC’s output, the proper voltage space vector is acquired. An analog of the flux linkages along with the characteristics of the torque generated, the estimated value of the generated torque, and the constant flux linkage are given by the HC. Six voltage vectors and two zero voltage vectors are generated by the voltage source converter. The mathematical relationship between flux, current, and the voltage of the stator and rotor are the factors coupled with the switching logic. The load angle is defined as the angle between the rotor and stator’s flux linkage. By combining the difference between the input voltage and voltage drop measured across the stator resistance in the direct control scheme, the stator flux linkage is evaluated, which is depicted as

(26) $$\beta_{d}=-\int \left(L_{d}-h_{d}\;Z_{s}\right)dx$$

(27) $$\beta_{q}=-\int \left(L_{q}-h_{q}\;Z_{s}\right)dx$$

#### 3.3.2. Fuzzy Controller

To maximize system performance concerning load needs, this method’s FC controls the generation and storage of electrical power. The power electronic converter’s duty ratio is adjusted using a controller called the FLC. The FLC is wielded to control the output voltage, dissipate surplus energy, and charge and discharge energy storage batteries. During fluctuating WS circumstances, the FLC is utilized to track and maintain a steady rotor speed. To conduct a specific electrical task on an electrical issue, the FL is a set of well-defined logic that is exhibited in the MF. Fuzzification, inference engines, and defuzzification are three of its operations. However, throughout the fuzzification process, the methodology has time-consuming trial and error difficulties with modifying the boundary values of MFs. Poor overall system performance is led by the incorrect selection of MF borders. For effective MF selection, the Weibull distribution-based chicken swarm optimization (WDCSO) algorithm was utilized in this methodology. The proposed controller is named EFLC.

**Fuzzification:** By the fuzzy reasoning mechanism, the measured inputs, called crisp values, are converted or translated by the fuzzification process into fuzzy linguistic values. The differences between speed command $\chi_{sc}^{*}$ and actual speed $\chi_{sc}$ and command torque $Q_{e}^{*}$ are the input and output of a speed controller in a PMSG vector control framework. So, the input taken is the speed error $E_{w}\left(k\right)$, the other input is the variable of speed error $E_{cw}\left(k\right)$, and the command torque $Q_{e}^{*}$ is replaced by t(k), which is utilized as the speed controller output. For every sampling time, the two inputs are:

(28) $$E_{w}\left(k\right)=\chi_{sc}^{*}\left(k\right)-\chi_{sc}\left(k\right)$$

(29) $$E_{cw}\left(k\right)=E_{w}\left(k\right)-E_{w}\left(k-1\right)$$

where $\chi_{sc}^{*}$ and $\chi_{sc}$ are the PMSG’s speed command and actual speed. During the fuzzification phase, an appropriate MF is required to alter the definite variables $E_{w}\left(k\right)$ and $E_{cw}\left(k\right)$ into fuzzy variables $E_{w}$ and $E_{cw}$. A subjective scheme element for FC is MF, and a triangular MF is utilized in this model. The universe of discourse is separated into seven fuzzy sets: negative big (NB), negative small (NS), negative medium (NM), positive medium (PM), positive big (PB), positive small (PS), and zero (Z). 

The WDCSO algorithm is utilized in this scheme to control the MF boundaries. Grounded on the hierarchy order along with the movement of a chicken’s swarm at the time of their food-searching actions is the bio-inspired algorithm named chicken swarm optimization (CSO). Diverse laws of motion are followed by every chicken. In the social lives of chickens, the hierarchical order is very important. Rooster movement, hen movement, and chick movement are the three steps of the CSO algorithm. The standard CSO delivers great performance, but there is sometimes a convergence problem to fix; therefore, in the rooster movement phase, this research methodology uses the Weibull distribution function rather than the Gaussian distribution function. 

First, all the populations are initialized and measured using the fuzzy rules membership function $\left(MF_{i}\right)$. 

After that, when analogized with the rooster movements having the worst fitness value, those with better fitness values can search for food in wider areas. Such movement is proffered in Equation (6):

(30) $$CC_{v,k}^{t+1}=CC_{v,k}^{t}\;\;\left(1+WB_{d}\right)$$

where the Weibull distribution is denoted as $WB_{d}$, the updated solution is signified by $CC_{v,k}^{t+1}$, and the current solution is symbolized as $CC_{v,k}^{t}$. Here, the torque minimization is measured as the fitness function.

The hens follow the group mate for foraging in the hen movement stage. Compared to the more submissive ones, the more dominant hens have the benefit of food competition. The hens’ movement is formulated in Equations (31)–(33):

(31) $$CC_{v,k}^{t+1}=CC_{v,k}^{t}+sl_{1}\;.\;\gamma \;.\;\left(CC_{r1,k}^{t}-CC_{v,k}^{t}\right)+sl_{2}\;.\;\gamma .\;\left(CC_{r2,k}^{t}-CC_{v,k}^{t}\right),\;\;\;\;\;\;\;\;\;\;\;\;\;\;\;\;\;\;\;\;\;\;\;\;\;\;\;\;\;\;\;\left(r_{1}\neq r_{2}\right)$$

(32) $$sl_{1}=\left(\frac{rg_{v}-rg_{r1}}{\left|rg_{v}\right|+\tau }\right)\;$$

(33) $$sl_{2}=\exp\left(rg_{r2}-rg_{v}\right)$$

where a random number evenly dispersed between 0 and 1 is denoted by γ. The rooster’s index is denoted as $r_{1}$, which is the v-th hens’ group mate, while the chicken’s index is signified as $r_{2}$. The algorithm randomly selects from the swarm, so random values are denoted as γ, rooster’s index is symbolized as $r_{1}$, which is the v-th hens’ group mate, while the chicken index is notated as $r_{2}$, and the fitness function is specified as $rg_{v}$.

**Figure 5:**
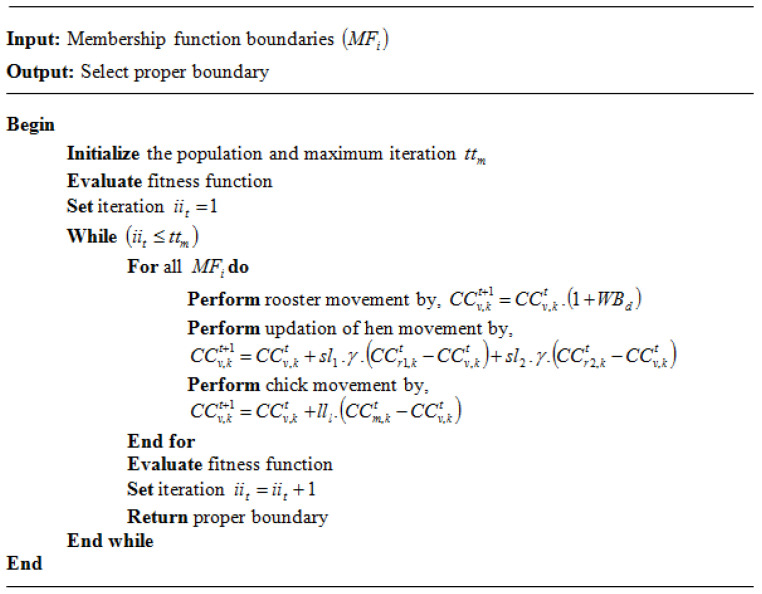
Pseudocode for the WDCSO.

Chick movement is the terminal stage. The foraging of chicks is made around the mother hens. The chicks’ updated method is described as

(34) $$CC_{v,k}^{t+1}=CC_{v,k}^{t}+\;ll_{l}\;\left(CC_{m,k}^{t}-CC_{v,k}^{t}\right)$$

where the m-th chick’s mother position is $CC_{m,k}^{t}$, such that m∈[1, N], $ll_{l}$ denotes a parameter representing the speed at which a chick runs behind its mother. Figure 5 illustrates the pseudocode for the proposed WDCSO.

**Inference Engine:** The knowledge base’s establishment is a vital point for the FC. To attain the control target, an expert control rule collection, called the knowledge base, is requisite. Using the PMSG behavior knowledge, the control rules are generated.

**Defuzzification:** The fuzzy reasoning mechanism outcome is transformed into the requisite crisp value by this procedure. The identified increment torque is denoted as a crisp value, which is estimated by Equation (35):

(35) $$dQ_{e}^{*}=\frac{\sum_{i=1}^{m}dQ_{er}\;f\left(dQ_{er}\right)}{\sum_{i=1}^{m}f\left(dQ_{er}\right)}$$

The torque $Q_{e}^{*}$ can be attained by utilizing Equation (36)

(36) $$Q_{e}^{*}\left(k\right)=Q_{e}^{*}\left(k-1\right)+dQ_{e}^{*}\left(k\right)*Q$$

where the FC’s output is depicted as $dQ_{e}^{*}$, corresponding to the r-th control rule, the input MF’s membership value is $dQ_{er}$, corresponding to the r-th control rule, $f\left(dQ_{er}\right)$ is the output MF’s membership value, and sampling time is proffered by Q.

## 4. Result and Discussion

In the proposed method, a prerequisite of the constant rotor presence is eliminated, and all the essential calculations are performed by keeping the stator as the reference. MATLAB/Simulink was used to simulate the suggested EFLC-centered torque ripple reduction in IPMSG performance, and the proposed methodology was put into practice. ‘’Table 1a,b show the wind speed and the IPMSG parameters, respectively’’.

### Performance Analysis

The torque ripple is examined here after the suggested EFLC was applied. Numerous factors were also examined, including the electromagnetic torque, the speed of the generator’s rotor, the three-phase voltage (*L*_abc_), the three-phase current (*h*_abc_), the outputs of the voltage on the d- and q-axes, the current flowing through the phase bridge converter, and the generator terminal. It is suggested that WDCSO’s performance can be examined in comparison to the popular particle swarm optimization (PSO), chicken swarm optimization (CSO), grey wolf optimization (GWO), and genetic algorithm (GA). This comparison is based on the fitness vs. iteration analysis.

**Figure 6:**
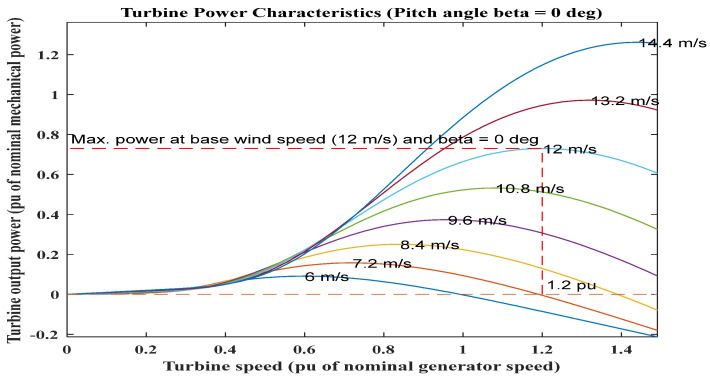
Graphical representation of mechanical power concerning turbine speed under fluctuating wind speed.

Figure 6 shows the rotor performance concerning rotor speed with erratic WS. The optimal power curve offers information on how to maximize energy harvesting under various WS. The turbine uses the controller to operate on the power curve under changeable WS circumstances to create maximum speed.

**Figure 7:**
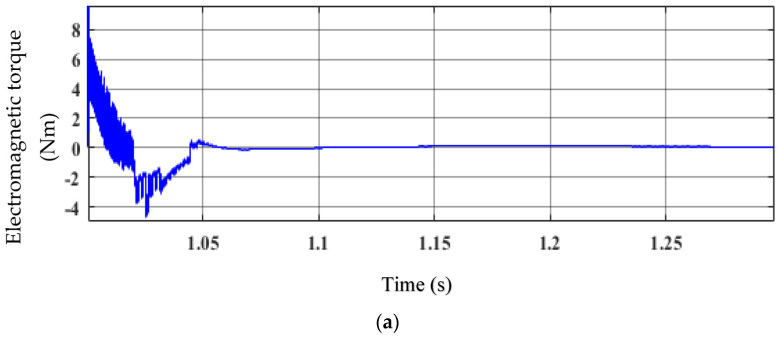

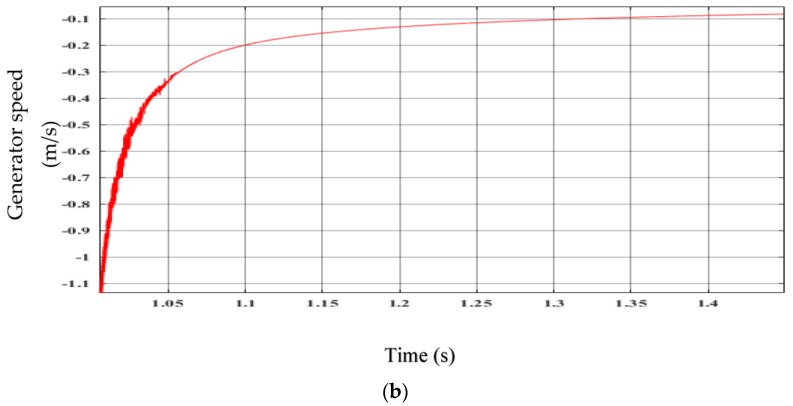
Performance of the EFLC: (**a**) electromagnetic torque and (**b**) generator’s rotor speed.

The effectiveness of EFLC in decreasing the torque ripple of an IPM synchronous generator when wind speeds are changing is demonstrated in Figure 7. The electromagnetic torque and generator rotor speed are depicted in Figure 7a,b. In Figure 7a, the torque ripple is significant and erratic from the beginning to 1.06 s. The torque ripple is reduced and reaches a steady state at t = 1.06 s. The generator’s rotor speed is shown in Figure 7b. At t = 1.055 s, the generator’s speed rose and achieved a steady state. Additionally, FLC is used to track the rotor speed and keep it consistent in order to maximize power. Additionally, it offers dynamic speed control in windy circumstances and with oscillating torque.

**Figure 8:**
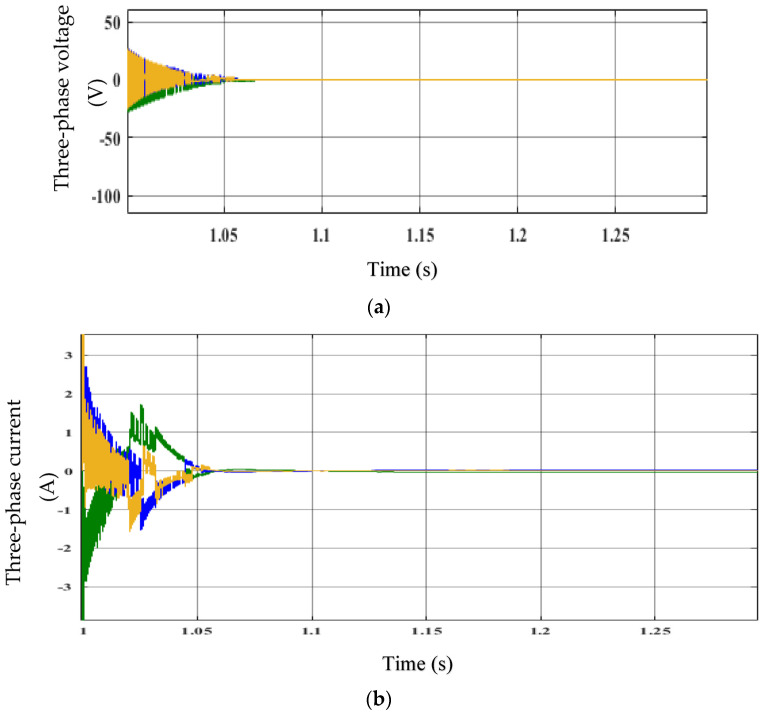
Performance of the EFLC: (**a**) three-phase voltage (*L*_abc_) and (**b**) three-phase current (*h*_abc_).

The three-phase voltage (*L*_ac_) and three-phase current (*h*_abc_) of the synchronous generator are shown in Figure 8a,b. The phase voltages (*L*_abc_) from the start of the graph to time t = 1.055 s reveal a strong ripple. The waveform’s ripple started to flatten and approach a steady state at t = 1.055 s. At t = 1.055 s, the current’s stability can be shown in Figure 8b. At this point, the ripple is at its lowest point. As a result, the controller demonstrates its capacity to control the generator’s voltage and current.

**Figure 9:**
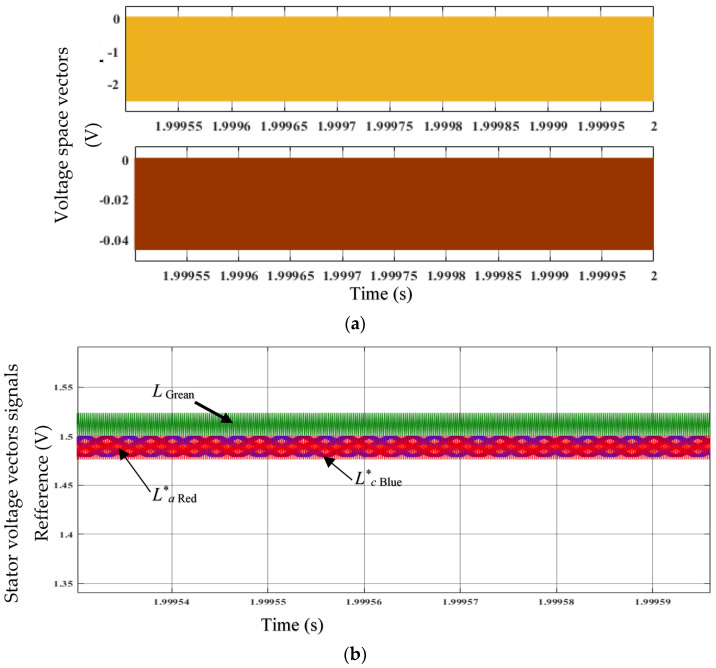
(**a**) Voltage space vectors L*_q_ and L*_d_. (**b**) Stator voltage vector signals L*_a_, L*_b_, and L*_c_.

The reference voltage space vectors L*q and L*d are shown in Figure 9a. The hysteresis controller gives the comparison of flux linkages and characteristics of the torque generated with the estimated value of the generated torque and the constant flux linkage, respectively. By choosing the voltage space vectors (L*q and L*d), the desired response of torque along with flux linkage is attained. When the estimated torque/flux exceeds the differential hysteresis limit, the torque/flux output decreases. When the estimated torque/flux drops below its differential hysteresis limit, the torque/flux status output increases. The differential limits, switching points for both flux and torque, are determined by the hysteresis bandwidth. Here, the torque output increased; therefore, the schema achieved the desired torque.

The stator voltage vector signals L*a, L*b, and L*c references are depicted in Figure 9b. These voltages are acquired by transforming the voltage space vectors L*q and L*d, created from hysteresis comparators, to feed the switching logic. Using switching logic, the correct stator voltage vectors are chosen to meet the torque and flux output requirements. The PWM module generates the gate control signals from these output voltage signals and feeds them to the three-phase bridge converter that rectifies the AC to DC to drive the GSC.

**Figure 10:**
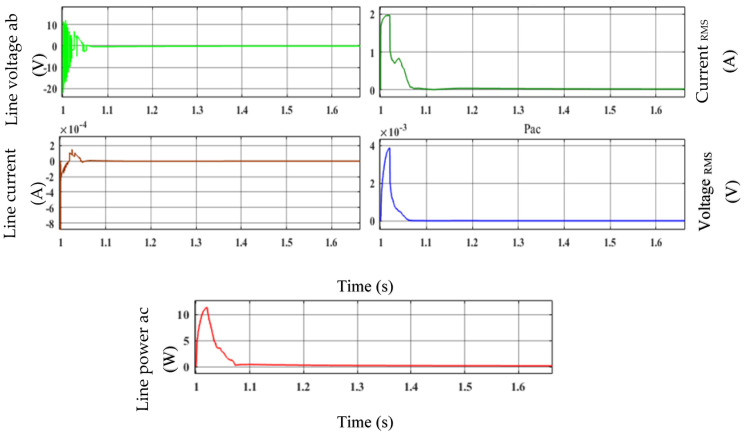
Performance of the EFLC: line voltage, line current, voltage RMS, current RMS, and power Pac.

The generator terminal is depicted in Figure 10. The line voltage ab, line current, voltage RMS, current RMS, and line power Pac reached a steady state at times of 1.06 s, 1.05 s, 1.07 s, 1.065 s, and 1.055 s, respectively. It makes sense that the EFLC regulates voltage and current to keep the generator running steadily despite changing wind speeds. Analysis of the voltage value reveals that the torque ripple is smaller than all of the temporal variations.

**Figure 11:**
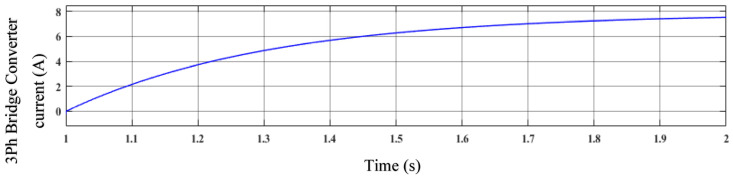
Three-phase bridge converter current.

The three-phase bridge converter current is shown in Figure 11. The three-phase converter is changed in accordance with the change in time. The current level has been significantly raised for now. The three-phase bridge converter operating with the suggested controller can function more effectively in this fashion.

**Figure 12:**
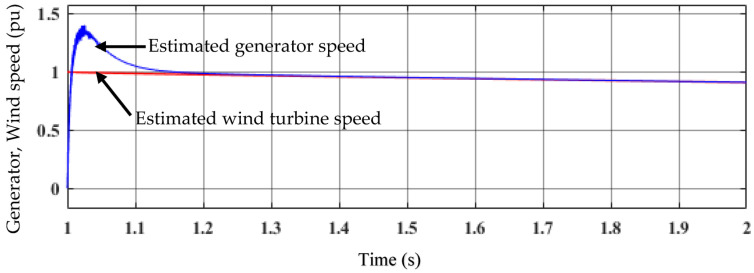
Estimated generator speed and estimated wind turbine speed.

The estimated generator and wind turbine speeds are shown in Figure 12. The measured variables, the generator voltages and currents, are used to estimate the generator speed. Both the d,q voltage and the d,q current are derived from stator currents (habc) and voltage (habc), respectively. The model reference adaptive system (MRAS) observer can be used to enhance the robustness and reliability of the drive system and to determine the generator speed without the need for a shaft-mounted mechanical sensor to avoid the drawback of the sensor. The sensor has several demerits, such as an increased number of connections between the generator and its controller, susceptibility to noise and vibration, the design complexity of the generator, and increased cost. The purpose of the MRAS observer is to calculate the PMG’s rotational speed. The figure shows that the estimated generator speed follows the estimated wind turbine speed quite well under varying wind conditions.

**Figure 13:**
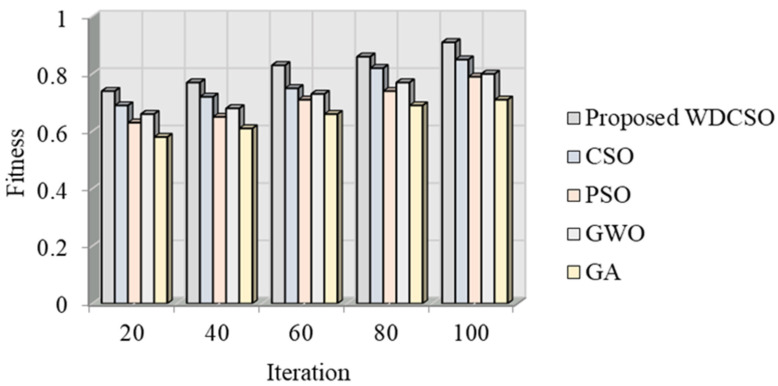
Fitness vs. iteration analysis.

Figure 13 offers a graphic depiction of fitness versus iteration. The suggested WDCSO is examined here using popular models, including the CSO, PSO, GWO, and GA algorithms. With 40 iterations, the suggested WDCSO algorithm has a fitness value of 0.77. The existing models have low fitness values when compared to the suggested model. Similarly to this, the suggested approach produced better results for different iteration numbers.

## 5. Conclusions

IPMSGs are widely used in the industry because of their high power densities and speed control capabilities. The decrease in TR of an IPMSG is a significant issue in the world of electrical machine design. The proposed model used the EFLC-based TR minimization system to resolve such issues under the change in WS. The HC and the FLC are both used in this model. To reduce the inaccuracy in the MF boundary selection, this model used the FLC’s WDCSO algorithm. The experimental analysis began with an explanation of the IPMSG and WT parameters. The study of the suggested controller’s torque reduction performance was then carried out, grounded on the voltage, current, generator terminal, electromagnetic torque, generator rotor speed, torque, and three-phase bridge converter current. According to the analysis, the proposed EFLC significantly reduces the TR. Based on the fitness analysis, the WDCSO was examined using the standard technique, demonstrating that the suggested methodology yields superior results. The performance of the proposed methodology can be improved in the future by using an advanced algorithm.

## 6. Limitations of Work

Though enhanced fuzzy logic is used to control the interior permanent magnet synchronous generator under variable wind speed, this means that the output of the control loop is not fixed at a specific value but is allowed to vary within a particular range. Overall, fuzzy logic has some major limitations, such as the handling of inaccurate data, the inherent inference of human thinking, and the fact that designing fuzzy logic takes time to reach precise data.

## Figures and Tables

**Table 1:** Parameters of WT and IPMSG.

S. No	Parameters	Values
(a)		
1	Nominal mechanical output power (W)	1.50 × 10^6^
2	Electrical generator’s base power (VA)	1.5 × 10^6^/0.9
3	Base WS (m/s)	12
4	Maximum power at base WS (p.u. of nominal mechanical power)	0.73
5	Base rotational speed (p.u. of base generator speed)	1.2
6	Pitch angle beta to exhibit WT power characteristics (beta ≥ 0) (deg)	0
(b)		
1	L_dc_	288 Vdc
2	Rated power	100 kw
3	Rated speed	12,500 rpm
4	Number of phases	3
5	Back EMF waveform	sinusoidal
6	Rotor type	salient-pole
7	Mechanical input	speed w
8	Preset model	No
9	Stator phase resistance Rs (ohm)	0.425
10	Inductances	[0.017415845761, 0.029268882377] H
11	Flux linkage	0.433 Wb
12	Pole pairs *p*	4
13	Initial conditions	[0,0,0,0]

## Data Availability

Not applicable.
